# Carbon Nanotube Sheet-Synthesis and Applications

**DOI:** 10.3390/nano10102023

**Published:** 2020-10-14

**Authors:** Megha Chitranshi, Anuptha Pujari, Vianessa Ng, Daniel Chen, Devika Chauhan, Ronald Hudepohl, Motahareh Saleminik, Sung Yong Kim, Ashley Kubley, Vesselin Shanov, Mark Schulz

**Affiliations:** 1 College of Engineering and Applied Sciences, University of Cincinnati, Cincinnati, OH 45221, USA; pujariaa@mail.uc.edu (A.P.); ngva@mail.uc.edu (V.N.); chenru@mail.uc.edu (D.C.); chauhadk@mail.uc.edu (D.C.); hudepord@ucmail.uc.edu (R.H.); ksy1357@pukyong.ac.kr (S.Y.K.); shanovvn@ucmail.uc.edu (V.S.); 2 Nanoworld Laboratories, University of Cincinnati, Cincinnati, OH 45221, USA; 3 College of Design, Art, Architecture and Planning, University of Cincinnati, Cincinnati, OH 45221, USA; salemimh@mail.uc.edu (M.S.); kubleyay@ucmail.uc.edu (A.K.)

**Keywords:** carbon nanotube, hybrid sheet, gas-phase pyrolysis, nanoparticles

## Abstract

Decades of extensive research have matured the development of carbon nanotubes (CNTs). Still, the properties of macroscale assemblages, such as sheets of carbon nanotubes, are not good enough to satisfy many applications. This paper gives an overview of different approaches to synthesize CNTs and then focuses on the floating catalyst method to form CNT sheets. A method is also described in this paper to modify the properties of macroscale carbon nanotube sheets produced by the floating catalyst method. The CNT sheet is modified to form a carbon nanotube hybrid (CNTH) sheet by incorporating metal, ceramic, or other types of nanoparticles into the high-temperature synthesis process to improve and customize the properties of the traditional nanotube sheet. This paper also discusses manufacturing obstacles and the possible commercial applications of the CNT sheet and CNTH sheet. Manufacturing problems include the difficulty of injecting dry nanoparticles uniformly, increasing the output of the process to reduce cost, and safely handling the hydrogen gas generated in the process. Applications for CNT sheet include air and water filtering, energy storage applications, and compositing CNTH sheets to produce apparel with anti-microbial properties to protect the population from infectious diseases. The paper also provides an outlook towards large scale commercialization of CNT material.

## 1. Introduction

Over the last two decades, carbon nanotubes (CNT) have attracted attention in the fields of sensors [1], filtering [2,3], energy storage [4], and other areas. There are various methods to synthesize CNTs, including arc-discharge [5], using plasma [6], laser ablation [7], chemical vapor deposition (CVD) [8], and the floating catalyst method [9,10,11,12]. Arc-discharge and the laser ablation process are high-temperature synthesis processes which can produce CNTs with fewer structural defects. Many nanotubes with little control over chirality can be produced using the arc-discharge method but the use of metallic catalysts needed for the reaction requires post-processing purification of the nanotubes. Nanotubes synthesized using laser ablation show high yield and low metallic impurities because of the tendency of the metallic catalyst to evaporate from the end of the tube once it is closed. Nanotubes obtained from this process are not uniform and contain some branching. Furthermore, the use of a high purity graphite rod and laser power makes the method expensive. The arc-discharge, laser ablation, and plasma methods produce powder nanotubes. Lower temperature synthesis processes, such as chemical vapor deposition, are commonly used due to better control of nanotube diameter, purity, length, orientation, and economical mass production of carbon nanotubes [13]. High-quality single-walled nanotubes (SWNTs) were prepared with the CVD method using a novel aerogel-supported Fe/Mo catalyst with high productivity. The number of nanotubes obtained per unit weight of catalyst showed a five-fold improvement compared to catalyst supported on Al_2_O_3_ powder. The strong interaction between the aerogel and catalyst and the high surface area of the support resulted in high catalytic activity [14]. The CVD method with a substrate produces forests of CNTs that can be pulled as a thin film and wrapped layer by layer into sheet in a secondary operation. The floating catalyst gas-phase pyrolysis method can produce industrial-scale carbon nanotube sheet directly by winding a web or sock of nanotubes onto a drum in a continuous process. High-quality, evenly distributed, aligned carbon nanotubes of 10-nm diameter can be prepared by the floating catalyst method. In one approach, to enhance the growth of nanotubes, benzene was used as the carbon source, ferrocene as the catalyst, and thiophene as the sulfur-containing additive [15]. The relation between the average nanotube diameter and thiophene concentration was studied [16]. The amount of sulfur added can vary the morphology of the nanotubes. An improved floating catalyst approach allowed lower growth temperature and closer control over the growth parameters to produce large quantity, high-quality, and low-cost single-walled carbon nanotubes. This method can be used to grow both single-walled and multi-walled carbon nanotubes (MWCNTs) and the yield is affected by the growth promoter which is a sulfur-containing additive [16].

The unique properties of CNT materials include good current carrying capability [17,18] good mechanical strength [19], high thermal conductivity [20,21], and others. The anisotropic heat conduction of CNT sheet, for example, makes it a promising candidate for firefighting applications. A smart garment concept was shown in a simulation to direct heat from the garment to an external cold sink, which lowered the temperature of the body [22]. Fire retardant properties of free-standing CNT sheets were also investigated and considered appropriate to prepare personal protective equipment (PPE) for firefighters [23]. Overall, the integration of CNT materials into textiles opens a wide area of applications [24]. The porous structure of very thin CNT sheet, or sheet containing particles, and the electrical conduction properties of CNT sheet also make it a promising candidate for energy storage for wearable electronics [25] textiles.

The porous nature of thin CNT sheet or sheet with particles, and the high surface area of CNTs, may be useful for air and water filtering applications. The conductive and antimicrobial properties of CNTs can be used for pathogen/virus capture and inactivation. To enhance the antimicrobial property, anti-viral nanoparticles (e.g., Ag, Cu, ZnO, etc.) can be integrated into the CNT synthesis process [26]. Sheet performance depends on the customization of the sheet, not just the intrinsic properties of the CNT. Customization can include altering the hydrophilicity and breathability of the sheet and integrating different types of nanoparticles (NPs) into the synthesis process to change the properties of the sheet.

This paper discusses the synthesis of CNT using the floating catalyst approach and describes a new approach to integrate metal nanoparticles with the CNT sheet to improve its properties. Furthermore, applications for this improved carbon nanotube hybrid (CNTH) sheet are discussed, such as filtering and energy storage along with associated manufacturing obstacles and commercialization.

## 2. Manufacturing CNT Sheet Using the Floating Catalyst Method

### 2.1. Carbon Nanotube Materials Synthesis

**Figure 1:**
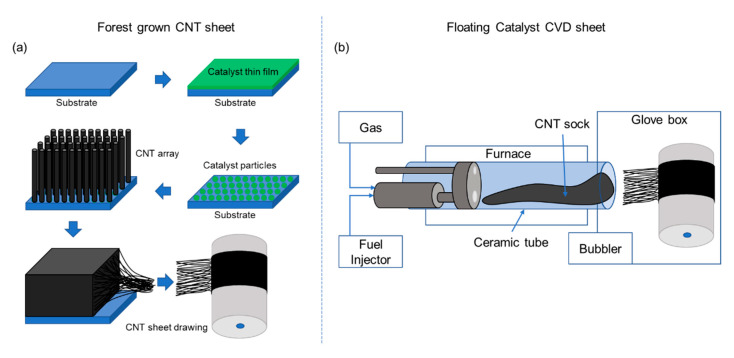
Schematic diagrams of carbon nanotube (CNT) sheet fabrication processes: (**a**) process of drawing forest-grown CNT into a sheet; (**b**) process of winding the floating catalyst chemical vapor deposition (FCCVD) CNT sock into a sheet.

In this study, we used the floating catalyst process to fabricate CNT sheet and carbon nanotube hybrid (CNTH) sheet. Figure 1 is a schematic diagram showing processes for making forest-grown CNT sheet and floating catalyst chemical vapor deposition (FCCVD) CNT sheet. Figure 1a is the process of manufacturing forest-grown CNT sheets. Usually, silicon is used as a substrate, and catalyst particles on which CNTs can grow are placed on it, and CNTs are vertically grown on it.

After the synthesis step, the vertically grown CNT is drawn to produce a CNT sheet. As can be seen in Figure 1a, it is necessary to go through various intermediate processes to manufacture the CNT sheet, which increases cost. Furthermore, since CNTs must be grown on a substrate, which must fit into the reactor furnace, it is difficult to produce a continuous and large amount of CNT sheet.

**Figure 2:**
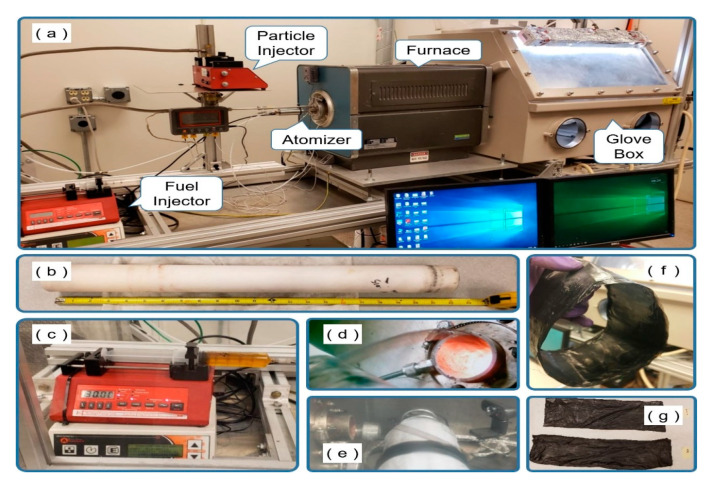
The fabrication process for carbon nanotube hybrid sheet: (**a**) experimental setup for CNT material synthesis; (**b**) short reactor (24-inch ceramic tube); (**c**) fuel, syringe pump, and atomizer controller which comprise the fuel injector; (**d**,**e**) the CNT sock forms in about 1 s and is wound up on the drum; (**f**,**g**) CNT sheet is peeled off the drum.

Figure 1b shows the process of manufacturing a CNT sheet using the FCCVD method. In this method, fuel including catalyst is injected into a tube in a high-temperature furnace to create a CNT sock. The sock exits the end of the tube and collected onto a rotating and translating drum to form sheet (see Video S1). This process is suitable for continuously synthesizing carbon nanotube materials stably in a relatively short time. It is also considered possible to scale up the synthesis process from the laboratory level to an industrial level. A floating catalyst reactor is shown in Figure 2a. A short ceramic tube with a 2-inch outer-diameter (OD) and 24 inches long is used as shown in Figure 2b. When running the reactor, the temperature of the furnace in the heating zone is around 1420 °C. There are three zones along the length of the reactor tube: the inlet zone, the hot zone, and the cooling zone or outlet. The length of each zone is about 8 inches and is defined by the insulation of the reactor tube. There is insulation at the inlet and outlet, whilethe center zone is the heating zone. Positive pressure inside the glove or harvesting box is maintained at between 0.15 and 0.25 inches of water to guard against air leaking into the system if there was damage. The ceramic tube and furnace hot zone lengths were adjusted to be as short as possible. The short process reduces the drag of the sock on the wall of the ceramic tube and this reduces breaking of the sock. Typically, a ceramic tube is about 36 inches for a 1.75-inch inner-diameter (ID) and 2-inch OD tube. The short tube used here also makes it possible to place more production equipment in a limited space due to the short reactor. For scale-up in the future, the short reactor is expected to reduce equipment manufacturing cost. The fuel used in the synthesis process is a mixture of ferrocene, xylene, n-hexane, and methanol. The fuel has an orange color due to the ferrocene (1% by weight) and is used in a syringe as shown in Figure 2c. The fuel is mixed during preparation, but to remain mixed, the fuel is also sonicated for 30 min before the experiments are conducted. As shown in Figure 2a,c, fuel is injected into the reactor by a syringe pump that feeds into a so-called fuel injector which consists of an ultrasonic atomizer and mixer where the fuel is mixed with Ar-H_2_ carrier gas. Fuel is delivered by the syringe pump into the atomizer and then into a mixer at a rate between 30 and 60 mL/hr. As the fuel enters the atomizer, it is broken into droplets that are micron-size using about 3 watts of power. The power of the atomizer is self-controlled and depends on the load of the fuel which may contain metal NPs. NPs can also be carried by the gases. When fuel is injected into the mixer through a needle inside the atomizer, Ar gas and H_2_ gas carry the fuel into the mixer, and when the fuel mixture leaves the mixer it forms a sock that is transported out of the reactor. The gas flow can vary depending on the process.

In a typical synthesis process, H_2_ gas flow is 100 SCCM, and Ar gas is used in the range between 100 to 2400 SCCM while using a video camera to continuously check the state of the sock coming through the outlet of the ceramic tube. Gases are included among various variables that determine the state of the sock, and we have produced the optimal CNT and CNTH sheet within the limit of reducing the use of gas as much as possible. The process must be carefully tuned to prevent breaking of the sock. To increase electrical and thermal conductivity and mechanical properties of the CNT, metal, ceramic, or carbon NPs are injected with the Ar gas during the CNTH manufacturing process through a separate route, using a custom particle injector. The Ar gas and H_2_ gas flowing through the fuel injector and particle injector into the glove box are controlled by mass flow controllers. When the fuel supplied from the fuel injector and metal NPs supplied from the particle injector are mixed in the mixer and injected into the high-temperature ceramic tube, the sock is formed and enters the glove box within 1–1.5 s. The sock leaving the outlet is wound up by a rotating drum, as shown in Figure 2d,e. When the wrapped sock is peeled from the drum and unfolded, it becomes a sheet, as shown in Figure 2f,g. Different particle injectors are being tested.

### 2.2. Integration of Nanoparticles in CNT Sheet

Carbon nanotubes have extraordinary properties and are increasingly being used in various fields of engineering and research. The sp^2^ structure enables CNTs to possess unique electrical and mechanical properties for a sheet. CNTs have highly stable shells, a high specific surface area, a large length/diameter aspect ratio, and a high Young’s modulus. The carbon shells can be wet by other materials without affecting the stability of the nanotubes [27]. Although individual CNTs have remarkable properties, CNT macroscale materials, such as sheet, have modest properties. Thus, there is an increasing demand to produce CNT macroscale assemblages with better properties. However, it is difficult to improve properties when the individual CNT is integrated into macroscopic CNT sheet. An approach to improve the properties of CNT sheet is to integrate nanoparticles (NPs) into the CNT sheet, which may selectively enhance its properties at the macroscopic level. In general, many types of NPs, including organic materials, metals, semiconductors, salts, etc., can be integrated into CNTs either by post-processing opening, wetting, and filling the nanotube shells, or by adding NPs during the synthesis process where chemical reactions can occur.

How different types of NPs interact with CNTs is complicated and not well understood. Several methods and approaches are proposed in the literature that involve combining various metals and alloys with CNTs. To combine CNTs and metals requires understanding the theory of wetting and solubility of CNTs with other materials. Substances that are used to wet and fill CNTs usually have a low surface tension energy with an upper limit of 200 mN/m [28]. This limit allows nanotubes to be wet by a few acids and organic solvents and can be used to fill the inner cavity of nanotubes [29]. Filling the inner cavity of nanotubes offers a possibility of producing quantum wires of different materials. Other nanoscale experiments could be performed to potentially produce new materials. Wetting of nanotubes enables coating and decorating the outer surface of the nanotube shells which allows the CNT to reinforce composite materials. Wetting of nanotubes is directly related to the surface energies of the liquid and solid surfaces of the nanotubes and with other substances. Wetting and filling of nanotubes are options for combining nanoparticles with CNTs. For wetting and filling to be successful, it is important to realize that substances which wet the nanotubes may not necessarily be useful in filling the capillaries of nanotubes. A few metals from the transition metal series, specifically group VIII systems, tend to wet CNTs and are extensively used as catalysts to improve the properties of CNTs. This is because these elements tend to have a higher solubility of their solid phase in the liquid phase, leading to precipitation of graphite on these metals [30]. Graphitic carbon formation on these transition metals has been extensively studied in producing high-quality nanotubes [31]. This is because at high temperatures, carbon forms a solid solution, and when the temperature is lowered, the solubility of carbon decreases, which allows the carbon to diffuse out to form more stable nanotubes. Along with the decomposition temperature, the rate of reaction and kinetics also play an important role in determining the precipitation of carbon. There is also a direct correlation between the growth of the nanotubes and the catalyst used. While producing the CNT sock using the substrate free-floating catalyst method, one of the main challenges is to improve the properties of the CNT sheets. One approach is to integrate nanoparticles into the synthesis process before CNT sheets are formed. The nanotubes can be combined with different NPs during the synthesis process. This combination process depends on the experimental conditions, the feedstock composition, and carrier gas composition. One of the most promising methods of partially wetting the nanotubes is by changing the feedstock composition. If the catalyst particle used contains low fractions of dissolved carbon, it leads to the partial wetting of the tube with metal particles [32]. A combination of a wide range of transition metal catalysts can be used based on their affinity towards carbon to produce sp^2^ crystalline carbon from solid solutions. Apart from group VIII metals, other carbide-forming transition metals, such as TaC, TiC, WC, HfC, and LaB_3,_ and those which have high coordination numbers are also capable of precipitating carbon to form graphene as well as nanotubes [33]. The outer surface of the carbon nanotubes can be wet by solutions of different metal compounds and then allowed to dry in a reducing environment. This allows the decoration of CNTs by different non-wetting metal particles [34]. Decorating the outer surface of the nanotubes also allows altering the properties of the nanotubes, especially improving their conductivity. There are many ways to do this depending on the experimental conditions used to produce carbon nanotubes, and the desired properties to improve. Apart from these further surface enhancements, techniques can mechanically treat nanotubes with other materials to improve their performance and properties, especially for semiconductor industries.

### 2.3. Materials and Methods

In this experiment, carbon nanotube sheets were synthesized using the floating catalyst chemical vapor deposition (FCCVD) method as discussed in Section 2.1. Fuel was delivered from a syringe pump to an atomizer. The particle injector distributed dry powder into the inlet of the reactor. The process to deliver the powder is still under development and will be described in detail in future publications. The furnace heated the fuel to 1420 °C to synthesis nanotubes. A sock of nanotubes exited the reactor tube and the sock was wrapped onto a rotating and translating drum to form a sheet that is hundreds of layers thick. The final sheet typically was in the range of 10 to 50 microns thick. The CNT sheet was characterized using standard instrumentation. Scanning electron microscopy (SEM) (FEI SCIOS, Waltham, MA, USA) and transmission electron microscopy (TEM) (CM-20, Philips, Andover, MA, USA) were used to characterize the surface morphology and nanostructure of the CNT sheet. For SEM characterization, no additional conducting coating was needed due to the high electrical conductivity of the CNT samples. Preparation of the TEM samples included dispersion of CNT samples in isopropyl alcohol (IPA). A tiny strand of the sample suspension was applied to a TEM grid and dried for one day for observation. Raman spectroscopy (Reinshaw inVia, Wharton Andech, UK) was used for structural characterization and to assess the quality of the CNT sheet. Tensile testing was performed using an Instron 5948 machine. A laser micromachining system (Oxford Laser A-Series, Didcot, UK) was used to cut the CNT sheet samples. The electrical conductivity of the sheet was measured using a four-probe technique.

## 3. Characterization of CNT Material

CNT use in different applications has kept growing since its first discovery in 1991 by Iijima [35]. Applications include fiber-reinforced material [36], water filtration, air filtration, energy storage [37], supercapacitors [38], textiles [39], structural health monitoring, and hydrogen sensors [40], to mention a few. The increasing integration of CNT material in various applications can be accelerated by doping CNT material with different elements of the periodic table to make it multifunctional. Multifunctional CNT hybrid material possesses enhanced properties relative to pristine CNT material, and may outperform traditional material in the intended application. Pristine CNT can be doped with metals [41], polymers, ceramics, and various chemicals [38,42] to meet various application needs. As there is an increasing trend in making CNT hybrid materials, characterization of the material becomes an important factor. The lack of scientific and industrial standards to characterize novel CNT hybrid materials has accelerated the development of new measurement techniques and new parameters for characterizing CNT hybrid materials [43,44]. In this section, we will investigate different characterization methods for CNT hybrid materials.

### 3.1. CNT Sheet Characterization Using Microscopy

**Figure 3:**
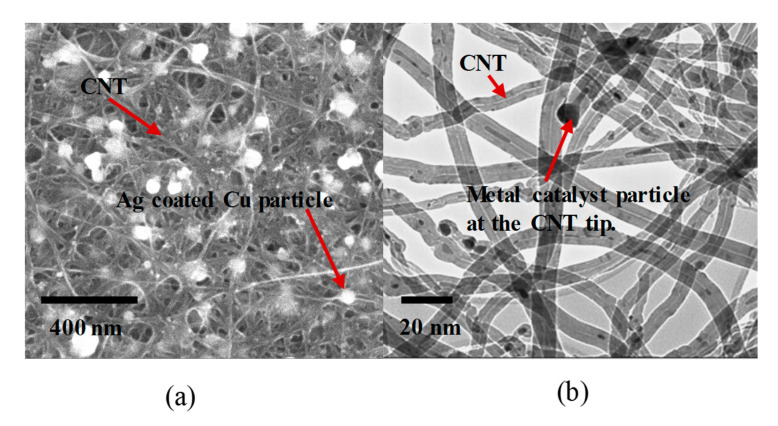
Scanning electron microscopy (SEM) and transmission electron microscopy (TEM) images. (**a**) Ag-coated Cu particle under SEM; (**b**) TEM image of multiwall CNT with a tip-growth model.

The scanning electron microscope (SEM) provides a visual understanding of CNT hybrid materials at the nanometer scale. An SEM employs a narrow beam of electrons that interacts with the subject, reflecting energy back which is collected to produce an image of the surface. Depending upon the type of CNT hybrid material, the sample is prepared accordingly for better surface imaging. A CNT hybrid material with a non-conducting polymer coating generally needs a sputtered metal coating to enhance the image resolution. SEM images provide visual information regarding the presence of surface impurities and CNT fiber alignment and structure. Figure 3a shows an SEM image of a CNTH material with silver-coated copper metal nanoparticles.

**Figure 4:**
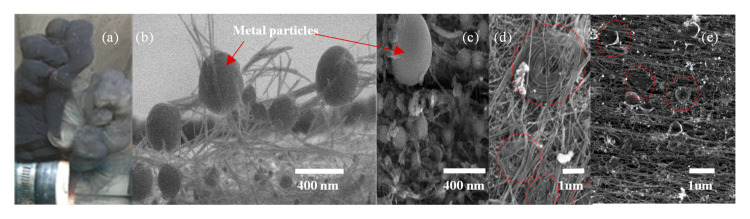
Carbon nanotube hybrid (CNTH) materials. (**a**) Pristine CNT (black sock) then particle injection turned on with Ag-Cu (grey sock); (**b**) metal particles “glue” bundles of CNTs together which increases thermal and electrical conductivity and possibly strength the CNT sheet; (**c**) CNT-Ag-Cu high-density nanoparticles (NPs); (**d**) CNT spiral bundles of CNT; (**e**) CNTH graphene cones.

The carbon nanotubes synthesized in this work use an iron catalyst and primarily exhibit the tip-growth mechanism, which is evident from the transmission electron microscopy (TEM) images in Figure 3b. In Figure 3b, higher density iron particles can be seen inside the low-density carbon nanotubes and at the tip end of the tubes. The CNT samples in this work were synthesized at 1400 degrees Celsius and the synthesized CNTs are primarily multiwall. However, to determine the exact number of walls in the synthesized CNTs, a high-resolution transmission electron microscope (HRTEM) is needed. Figure 4a–e shows CNTH samples from our existing reactor. Ag-coated Cu spheres were formed from thin platelets injected into the synthesis furnace (Figure 4b,c). Other material forms, such as cones and spirals, can be synthesized probably due to the metal NPs acting as catalyst particles (Figure 4d,e).

### 3.2. Raman Spectroscopy

**Figure 5:**
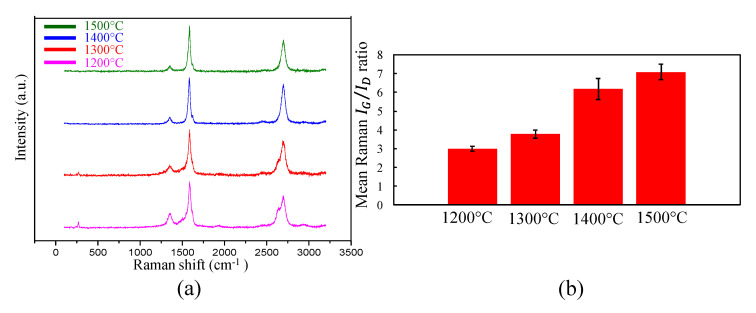
Raman spectroscopy: (**a**) the Raman scan of a CNT sample; (**b**) the *I*_G_/*I*_D_ ratio of the CNT samples. This testing shows that higher temperatures increase the crystallinity of the CNTs. Changing other process conditions, such as fuel injection rate, also affects the Raman spectra and can produce higher *I*_G_/*I*_D_ ratios.

Raman spectroscopy depends on the Raman effect, which is the scattering of light on interaction with the sample. Based on the energy absorbed or given up by the molecule of the sample upon interaction with the incident photon, the phenomenon of photon interaction is classified as stokes scattering or anti-stokes scattering. Raman spectroscopy provides information about the sp^2^ bonds on the CNTs. The presence of the sp^2^ bond is depicted by the presence of a G-band peak at ~1580 cm^−1^ of the Raman scan on the sample (Figure 5). The presence of symmetrical disorder in the hexagonal carbon structure of the CNTs is depicted by the disorder-induced D-band peak which appears at ~1345 cm^−1^. The quantification of the disorder in the hexagonal carbon structure of the CNT is calculated by taking the ratio of the intensity of the G-band (*I*_G_) to the intensity of the D-band (*I*_D_). A higher *I*_G_/*I*_D_ ratio indicates fewer defects in the CNT hexagonal structure. The presence of a radial breathing mode (RBM) peak at ~150 cm^−1^ indicates the presence of single-wall CNTs in the scanned sample. Figure 5a shows a typical scan from Raman spectroscopy of a single wall CNT. Generally, >10 scans are taken per sample and the mean of the I_G_/*I*_D_ ratio of the scans represents the *I*_G_/*I*_D_ of the sample. Figure 5b shows the mean *I*_G_/*I*_D_ ratio of 12 scans for CNT sheets synthesized at different temperatures.

It can be observed in Figure 5a that as the synthesis temperature increases, there is the loss of the RBM mode, which indicates higher production of multiwall CNTs than single-wall CNTs for high temperature synthesis. Moreover, as the synthesis temperature increases, there is an improvement of the hexagonal CNT structure which is evident from the increase in the *I*_G_/*I*_D_ ratio with an increase in synthesis temperature, as shown in Figure 5b.

### 3.3. Tensile Strength Testing

**Figure 6:**
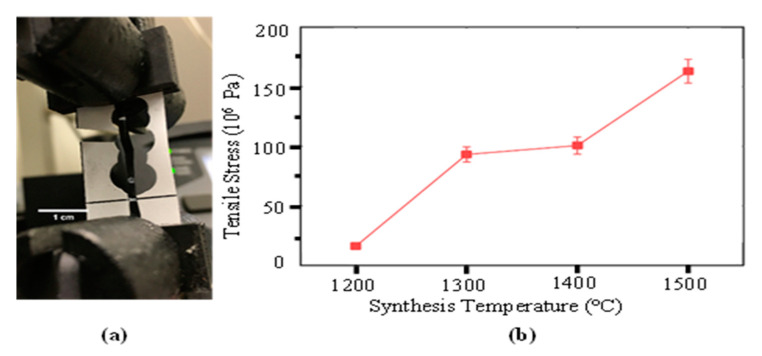
Tensile strength testing of CNT pristine sheet. (**a**) Tensile strength testing using an Instron tensile test machine with a paper frame to hold the sample. (**b**) Mean tensile strength of CNT sheets synthesized at different temperatures.

Tensile testing of the CNT sheet samples was performed using an Instron 5948 machine. There were variations in measuring the tensile strength of the CNT sheet depending on the cutting, thickness, and size of the sheet. Here, the sheets were cut in strips 2-mm wide × 20-mm long using a laser micromachining system. The samples were bonded to a paper frame [44] (Figure 6a). The tensile strength of CNT pristine sheets synthesized at different temperatures is shown below. The temperature was measured in the furnace chamber surrounding the ceramic tube. The temperature inside the ceramic tube will be lower than the temperature in the furnace chamber. The gas injection and the fuel vaporizing cool the gas inside the tube. The strength of the sheet can be increased in several ways, including increasing the winding speed of the sheet, which tends to partially align the CNT, using thinner samples, and by densifying the sheet using a rolling mill or other ways. Mechanical testing of the CNTH sheet will be performed in the future.

### 3.4. Electrical Conductivity

**Figure 7:**
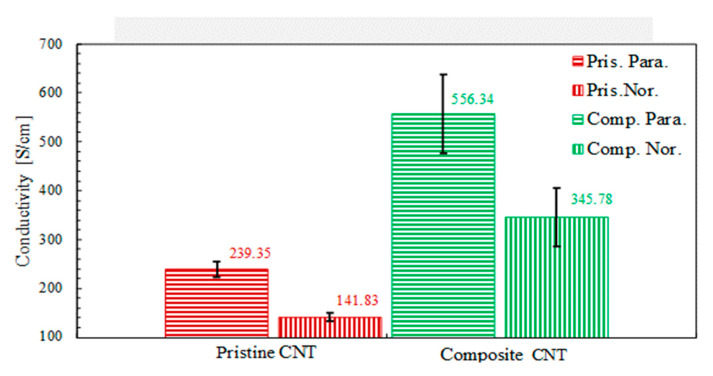
Electrical conductivity in two orthogonal in-plane directions of the 2D CNT sheet. The composite CNTH sheet has a small percentage of Cu NPs.

The electrical conductivity of the CNT sheets was measured using the four-point potential measurement technique. CNT sheets synthesized by the floating catalyst method and by the substrate based chemical vapor decomposition method are primarily anisotropic (have different physical properties at least in two different directions). Therefore, a linear four-probe arrangement produces an average measure of the electrical conductivity of the anisotropic CNT sheets. A square four-probe arrangement measures the electrical conductivity of the anisotropic CNT sheets [44,45] in two directions. Figure 7 shows the electrical conductivity of the CNT–metal composite material with a small percentage of metal NPs. It is difficult to characterize the number of NPs in the sheet. The NPs increased the electrical conductivity and reduced the electrical anisotropy of the material.

## 4. CNT for Energy Storage Applications

**Figure 8:**
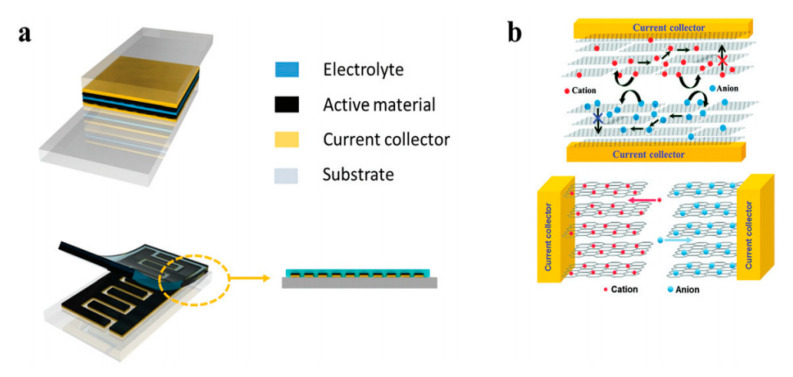
Electrical energy storage: (**a**) the basic supercapacitor design (above: stacking plane; below: interdigitated pattern); (**b**) schematic of ion movement during the process of charging and discharging—the lower figure shows higher efficiency for the ion diffusion. Reproduced from [46] with permission from the Royal Society of Chemistry, 2020.

The discovery of carbon nanotubes has stimulated immerse interest among researchers in academia and industry for next-gen energy applications. Although many different CNT synthesis methods have been developed, the problems inherent in CNT synthesis have become more prominent along the way, such as very limited batch-process throughput, low purity, and low production yield. Floating catalyst CVD (FCCVD) has the potential to reduce these problems. Additionally, due to the flexible reactor design and unique continuous FCCVD process, the porous, flexible, freestanding films as-synthesized possess many advantages, which include good electrical properties, high mechanical reliability, and ease of handling. As an example, the CNT sheet has a large surface area and can be used to form the electrodes of a compact supercapacitor (Figure 8).

Charge storage has three mechanisms: the first involves an electrical double layer (EDL) capacitance, which stores charge on the surface of the electrode reversibly. The second is called the Nernstian process, also called a non-capacitive Faradaic process, which mainly follows the Nernst equation to describe the transfer of the localized valance electrons. The third is called the capacitive Faradaic process, which involves a pseudo-capacitor, the capacitive pathway based on the transfer of the delocalized valence electrons [47].

**Figure 9:**
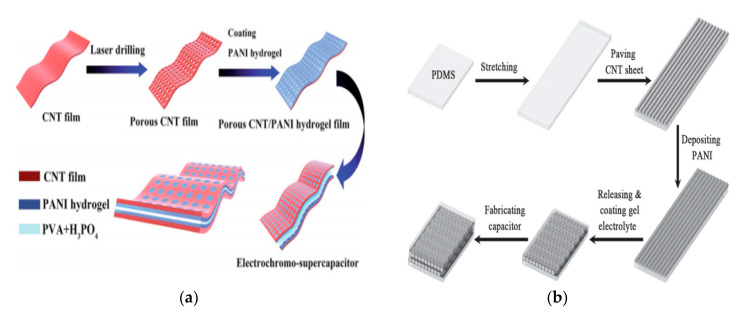
Device design: (**a**,**b**) different schematic processes for the fabrication of flexible CNT supercapacitors, both methods use polyaniline (PANI) as the active material. (**a**) Reproduced from [49] with permission from the Royal Society of Chemistry, 2020; (**b**) reproduced from [50] with permission from Wiley-VCH GmbH, 2020.

Transition metals and conductive polymers have been widely used as pseudocapacitive materials, which can provide capacitive Faradaic charge storage from the transfer of delocalized valence electrons [47]. Electrically conductive polymers (ECPs) are called synthetic metals due to their intrinsic electrical conductivity, which results from the full delocalization of π electrons on the long-chain aromatic polymer backbone [48]. Three major conducting polymers for active materials, i.e., polyaniline (PANI), poly (3,4-ethylene dioxythiophene) (PEDOT), and polypyrrole (Ppy), have demonstrated great potential in the application of energy storage owing to their high conductivity and superior capacitive properties. Conductive polymers paved the way for the exploration of CNT-conducting polymer composite materials. A supercapacitor device is illustrated in Figure 9.

Several groups have used CNTs and related hybrids for supercapacitors and battery applications, either in sheet form or in fiber form. Although CNT-based energy storage shows encouraging results, one of the main challenges remaining is preventing the reduction in the surface area during the fabrication of the device. The reduced surface area has an adverse effect on the electrochemical performance of the electrodes, as well as providing limited space for loading active materials into the sheet, and it causes poor electrolyte diffusion [51].

## 5. CNT Filtering Applications

Carbon nanotube bundles are held together by van der Waals forces and these entangled nanotubes in a thin sheet create a porous structure which can be used to trap and immobilize virus/pathogens. Thick CNT sheets become almost impermeable. The inner core diameter of the carbon nanotube and nanotube growth can be controlled by the catalyst particle [52,53] and the core diameter is in the size range of many viruses, proteins, and other biological macromolecules [54]. The membrane can be formed by growing CNTs on a substrate and the voids must be treated with a filler material. Carbon nanotube membranes with a pore size of 2 nm were grown on a silicon chip. To produce a void-free membrane, spaces between the CNTs were sealed with silicon nitride. These membranes transported water and gas extremely fast which was supported by molecular dynamic simulations [55]. Vertically aligned carbon nanotube (VA-CNT) membranes showed an advantage over CNT membranes with filler material by eliminating the spaces between CNTs grown by CVD. No filler materials were used because interstitial pores were in the range of CNT pore size, hence more permeation area was available and resulted in higher porosity (~20%) [56]. Purification steps, such as heat treatment and acid treatment, remove the amorphous carbon and introduce functional groups which will improve the interaction of CNTs with other polymer and solvents. Chemical functionalization at the entry of CNT cores offers a nanoscale scaffolding to allow “gatekeeper” chemical interactions which improve both flux and selectivity of the separation application [57]. The porosity of CNTs can be improved by oxidation to attach functional groups such as COOH and OH. A large amount of oxygen-containing functional groups present at the surface of oxidized multiwall CNTs (MWCNTs) makes nanotubes more easily dispersed in the water and provides binding sites to interact with metal ions, which can be completely and easily adsorbed [58]. The interaction of iron with oxidized CNTs was found to be higher compared to raw CNTs because of the reduced diameter and fewer impurities due to the presence of an oxygen-containing group [59]. Modified MWCNT treated by a mixture of HCL and H_2_O_2_ and nitric acid showed 97% filtering efficiency for chromium. The removal efficiency of the modified filter was higher due to the large surface area and small nanotube diameter [60].

Carbon nanotubes have emerged as a promising solution for water purification due to their remarkably large surface area and their ability to be chemically functionalized to increase their affinity towards target molecules [61]. Magnetically aligned membranes showed a smoother surface and shearing along the axis of the applied magnetic field. The alignment was uniform across the surface and through the depth of the membranes [62]. A macroscopic hollow cylinder, designed by the spray pyrolysis method, successfully filtered heavy hydrocarbons from petroleum and bacteria from drinking water. The advantage of these kinds of filters over conventional filters is their reusability. The filters can regain their full filtering efficiency after applying a process such as ultrasonication and autoclaving [63]. Carbon nanotube membranes of sub-nanometer diameter provided efficient water desalination in reverse osmosis [64]. A CNT–metal filter developed by growing CNTs on the metal filter using the CVD method showed high filtration efficiency [65]. A plasma-modified ultra-long CNT (UCNT)-based porous membrane is used for the removal of salts, organic matter, and metal nanoparticles. Water treatment techniques, such as reverse osmosis, require high pressure and have limited mobility. UCNT-based membranes overcome these limitations as only a small amount of pressure is required to achieve sufficient water flux to desalinate water and remove organic and microbes using the free energy of adsorption for desalination. The adsorption capacity of these membranes was recovered by rinsing the membranes with tap water [66]. A multi-walled carbon nanotube/aromatic polyamide nanocomposite membrane rejected salts and humic acid by factors of 3.17 and 1.67, respectively, compared to the membranes without nanotubes. However, membrane permeability decreased by 6.5%, which can cause adsorptive fouling due to increased hydrophobicity and can degrade membrane performance in the long term [67]. A polyacrylonitrile (PAN) ultrafiltration membrane filled with hydroxyl-functionalized multi-walled carbon nanotubes improved the transport properties and the mechanical stability of the membrane. The inclusion of well-dispersed nanotubes increased the polymer solution viscosity and lowered the formation of macro voids [68]. A polymer-CNT composite membrane synthesized using interfacial polymerization was suitable for both aqueous solution-based nanofiltration and solvent resistant nanofiltration. Nanotubes were dispersed in an aqueous solution containing poly(ethyleneimine) (PEI) monomer, and the outer surface of the nanotubes was functionalized using hydrophobic and hydrophilic groups through microwave treatment. The base of the polymer membrane developed solute selectivity and a low resistance pathway at the interface and was created by the nanogaps at the outer surface of the nanotubes. The solvent fluxes were one order of magnitude higher than for commercial membranes [69]. Granular activated carbon (GAC) has a large surface area and a strong affinity for organic matter. The combination of a GAC filter as a pre-treatment followed by flocculation resulted in better removal of organics, a lowered flocculant dose, and helped in reducing biofouling of the membrane [70].

Volatile organic compounds (VOCs) present in the atmosphere lead to severe health issues. Carbon sorbents have high removal efficiency and are cost-effective. One study suggests that amorphous carbon provided more sorption capacity for organic chemicals and the curvature and topological defects affect the sorption process [71]. Pressure drop and filtration efficiency are two key factors of filter performance. A high pressure drop indicates high energy consumption which promotes high filter efficiency. There are a few mechanisms for particles to collide with the surface and deposit on the filter, such as diffusion, inertial impaction, and an interception. Inertial impaction and interception come into play when there is an increase in particle size. On the other hand, Brownian diffusion occurs when there is a decrease in particle size. In the case of intermediate particle size, no mechanism is dominant and two or more mechanisms occur simultaneously, which leads to maximum particle penetration through the filter, hence lower filter efficiency. Filters coated with a polytetrafluoroethylene (PTFE) membrane showed “v”-shaped efficiency curves for particle sizes from 10 to 300 nm at face velocities from 0.3 to 15 cm/s [72]. Nanofiber filters were synthesized by electrospinning nylon nanofibers on a microfiber substrate used in nano-aerosol filtration. It was found out that the flow was dominated by diffusion, considering that larger airflow reduces the retention time of the aerosol inside the network and hence lowers the time for the aerosol to deposit on the fibers. Due to the continuous loading of the submicron aerosol, the filter clogged and elevated the pressure drop. It is suggested that in high-efficiency filtration applications, stacking up low basis weight nanofiber layers to create a multi-layer filter is more effective than using a single high basis weight nanofiber layer filter [73]. In continuous loading of the aerosol on the filter, there will be less available pore space because formerly available free space is taken up by the aerosol, which results in lower permeability and higher pressure drop. This will create a serious problem in the upstream region. A “skin region’ will be formed at the upstream end of flow due to the packed dense layer of aerosols. A dual-layered arrangement with microfiber upstream and nanofiber downstream was proposed. The study showed that upstream microfibers filtered the incoming aerosol, which reduced the degree of loading in the downstream nanofibers. The microfiber layer has a higher dust holding capability, so the aerosol deposition does not induce extra pressure drop. Now, there will be a uniform incoming aerosol layer at the downstream nanofiber which reduces the skin region [74]. A novel filter was prepared by drawing aligned nanotube sheets onto the polypropylene melt-blown sheet and embedding CNT layers in a cross-ply structure. The three-layer CNT cross-ply filter achieved the highest quality factor and met the high-efficiency particulate air (HEPA) filter criterion [75].

The recent outbreak of COVID-19 prompted a global response to biological aerosol exposure. SWNTs presented strong antibacterial activity. Direct interaction of bacteria with high purity, narrow-diameter nanotubes caused severe damage, and cell inactivation [76]. A comparative study of the antibacterial capability of highly purified SWNTs and MWCNTs showed that SWNTs have stronger antibacterial activity, indicating that the diameter of CNTs is a key factor in the inactivation of bacteria. A smaller-diameter nanotube can ease the penetration of nanotubes in the cell wall. Other factors that attributed to this increase were a large contact surface area and unique electrical and chemical properties [77]. A combination of nisin with a CNT-coated filter provided efficient bacterial capture and inactivation in one filtration step. Nisin adsorption on MWCNTs decreased the hydrophobicity of the surface, hence the greater the nisin adsorption, the lower the hydrophobicity [78]. The SWNT filter showed strong inactivation of bacterial and fungal aerosols in both indoor and outdoor environments. Filters with high CNT loading lowered both bacterial and fungal aerosol concentrations [79]. An electrochemical MWCNT microfilter showed removal and inactivation of the virus and bacterial pathogens at a low voltage of 1–3 V. The advantages over conventional filters include the antimicrobial activity of MWCNTs, high surface area, and small pore size which can sieve bacteria and remove bacteria by depth filtration, availability of large electrochemically active sites, and increased corrosion stability [80]. A CNT-metal oxide membrane composed of CNTs and silver nanoparticles improved the anti-bacterial property of the membrane. Silver acted as a welding agent to bond the CNTs together and transformed the membrane from hydrophobic to hydrophilic. The porosity of the membrane was also increased by increasing the silver content [81]. A novel CNT-based facepiece respirator was tested on a manikin-based system. Filters with high CNT loading improved the filtering performance and it was observed that CNT filters have higher biological aerosol particle filtration efficiency than total aerosol particles [82]. A detailed discussion about the integration of CNTH sheet in textiles, as well as its application in heat resistant materials, wearable electronics materials, and first responder gear, was discussed [83].

## 6. Safety of CNT Sheet

The Environmental Protection Agency (EPA) classified sheets with long CNTs (made from the floating catalyst method) as “articles” and not “particles,” meaning they are too large to be inhaled or absorbed by the skin [84]. Another organization also mentions CNT sheet as being safe [85]. CNT pristine sheet was tested for particle release [41], where particles were monitored using a personal ultrafine particle (UFP) counter (PUFP C110, Enmont LLC, Cincinnati, OH). No cleaning or post-processing of the sheets was performed. No particle release above the background level was measured during the flexing of the sheet.

When a CNT sheet is tested in a tensile test machine, the CNT sheet fails by the bundles of CNT slipping apart. Individual CNTs are flexible and strong, and it is believed that CNTs do not fracture or release particles when they fail. CNT sheet that is rubbed can transfer CNT bundles to shear surfaces. Thus, in textile applications, a veil or outer layer can be used with CNT sheets for safety.

## 7. Scaling up Manufacturing

Manufacturing of CNT sheets is done at grams per day levels at universities [86,87,88] and larger levels at a few industries [89,90]. However, there is no very large-scale manufacturing of CNT sheets today. A theoretical (speculative) new reactor scalable to large sizes is being designed, thus possibly reducing the problems of cost and throughput of CNT material. Limited testing indicates that the reactor yield is proportional to the cross-sectional area (square of diameter) of the fuel injector tube in the reactor. Presently ¼- and 3/8-inch ID extender tubes are used in a 2-inch OD reactor tube and can produce about 7.5 g of CNT sheet per day. A new furnace might use injectors that can be scaled up to a 2.5-inch diameter with a corresponding larger furnace. Scale up of output for a 2.5-inch injector tube is (2.5/(1/4))^2^ = 100. Thus, the new reactor might produce 100 × 7.5 = 750 g/day (this prediction must be verified) of CNT sheet. Assuming 260 work days/yr, the scale-up reactor could produce 195 kg/yr. About six reactors (in a small manufacturing facility) could produce a metric ton (1000 kg) per year of CNT sheet. Manufacturing cost is mainly due to capital (amortized over 7–10 years) and labor to operate the reactors. Raw materials, electricity, and facilities are smaller costs. Reactors larger than the example shown would be needed to further reduce costs. The new reactor offers a commercial opportunity to manufacture CNT and CNTH sheets. The commercial opportunity becomes greater if end products (e.g., air filters or apparel, water filters, toxic gas filters, textiles, PPE, electrical shields, conductors, etc.) are manufactured instead of just CNT sheets. The reactor design is based on the scale up of a research reactor using a different configuration of the furnace. The reactor design is compact and multiple reactors can be put in a small factory, and two operators might monitor the condition of the reactors using a central computer system. The reactors must be cleaned daily and this adds a labor cost to the manufacturing.

## 8. Summary and Conclusions

This paper described the synthesis of CNT sheets using a substrate-grown forest and by a floating catalyst method. Examples from the literature were reviewed. A new method was also presented for integrating nanoparticles via a custom particle injector to produce a carbon nanotube hybrid sheet in a one-step floating catalyst synthesis process. This CNTH sheet is multifunctional and has good electrical and mechanical properties. The integration of nanoparticles depends upon the wetting and solubility of the CNTs with other materials. Wetting of the nanotubes enables decoration of the nanoparticles on the outer surface of the nanotube which can be helpful in many applications such as composite materials. Chemical functionalization of CNT sheets is another way to improve the interaction with particulates and adsorption properties for filtering applications. Due to the great potential of CNTs, companies are investing in their various applications in textile, wastewater treatment, air filtration, sensors, and others. There are certain drawbacks as CNTs at the macroscale are expensive and the synthesis process has a low yield. To overcome these barriers, the need for the development of larger reactors, improving properties by improving the synthesis process, and better understanding the science behind CNT nucleation and growth are important.

## Supplementary Materials

The following are available online at https://www.mdpi.com/2079-4991/10/10/2023/s1, Video S1: Synthesis of a CNT rope.
